# Education as a Driver to the Demographic Dividend

**DOI:** 10.1111/1751-7915.70100

**Published:** 2025-02-11

**Authors:** Anderson Rocha de Jesus Fernandes, Bernardo Lanza Queiroz

**Affiliations:** ^1^ Centro Federal De Educação Tecnológica De Minas Gerais (CEFET‐MG) Belo Horizonte Brazil; ^2^ Universidade Federal De Minas Gerais (UFMG) Belo Horizonte Brazil

**Keywords:** demographic dividends, education, population age structure

## Abstract

Demographic changes, particularly population aging, influence societal and economic development. While often seen as a challenge, aging also presents opportunities through demographic and educational dividends. This commentary discusses how improvements and expansion of education might generate a demographic dividend. Education boosts productivity and well‐being, helping mitigate the economic pressures of aging. However, realising these benefits requires equitable, high‐quality education and labor market reforms to support diverse and aging workforces. Education is pivotal for sustainable development, especially in low‐ and middle‐income countries. It addresses aging‐related challenges, maximises demographic dividends, and fosters long‐term inclusive economic growth.

Demographic dynamics are an important factor for understanding the social, economic, political, and cultural aspects that govern human interactions over time and space (Lee, 2003; Lutz, 2021; Reher, 2011). Changes in population age structure might impact society and economy in different dimensions. For some time, population aging was considered an onus as a larger share of older adults would be supported by a smaller number of working‐age individuals (Cutler et al. 1990).

More recently, a large body of literature (Dörflinger and Loichinger, 2024; Mason and Lee, 2006), showed that population aging should be seen as an opportunity and a potential to economic growth and development. Two main benefits are possible: the first one is associated with the rapid increase of working‐age individuals compared to the dependent population, during the process of change from younger to older, representing a potential to boost economic growth. Eventually, it dissipates when the working age population grows older. The second is related to changes in saving behaviours by individuals that face their own process of aging, and its magnitude depends on how wealth is accumulated (Mason and Lee, 2006).

In addition, demographic dividends have labor market implications, since labor supply is a life cycle issue and linked to working characteristics: education, (in)formality, occupational status, wages, health, and leisure (Berkman and Truesdale, 2023; Prskawetz and Hammer, 2018). It is also related to the sustainability of public pension programs as several policy options to rebalance pension system involve different types of structural reforms (rising contributions, retirement ages, funding aspects) and behavioural changes towards labor market transition, i.e. measures that creates incentives for individuals to stay longer in the workforce.

However, working longer is not an option for all. Several factors must be considered to understand the aspects that lead people to stay in the labor force. Education plays an important role (Lutz and Kebede, 2018): more educated individuals are more productive, have more cognitive occupations that pay better wages and a set of conditions related to higher propensity to longer participations in the labor force (Bratsberg et al. 2023; Fernandes and Queiroz, 2024; Gordo and Skirbekk, 2013; Loichinger, 2015; Loichinger and Prskawetz, 2017; Marois et al. 2019). Individuals with higher levels of education are also more prone to healthier behaviours and to have more access to leisure. Human capital accumulation has the potential to increase productivity at all ages, and maintain cognitive capacity later in life, having important impacts on economic dynamics as populations move towards an older age structure (Skirbekk, 2004; Fertig et al. 2009; Deming, 2022; Schneeweis et al. 2014).

It is relevant to promote improvements in educational attainment, specifically in low‐ and middle‐income countries, that face significant levels of socioeconomic inequalities that might impact the capitalization of the demographic dividend and potentialize the negative impacts of the aging process (Baerlocher et al. 2019; Crespo Cuaresma et al. 2014; Fernandes and Queiroz, 2024; Kotschy and Sunde, 2018; Loichinger, 2015; Lutz et al. 2019). The effects of education can be assessed from its consequences on total productivity and macroeconomic development.

Crespo Cuaresma et al. (2014) dismiss the importance of a changing age structure in the productivity of the labor force. It would be explained only by changes in the educational composition. The authors argue that the demographic dividend is, in fact, an educational one. Lutz et al. (2019) reaffirm this argument and highlight the importance of human capital for increasing income per capita. Only with progress in education, a higher number of individuals in the workforce (age structure) would effectively produce economic growth.

Other studies point out the importance of education combined to the role of age structure to the demographic dividend. Rentería et al. (2016) show that both age and education are relevant to determine the variation of economic support ratios (ESRs) for Mexico and Spain, even though the effects of age tend to become negative through time. Kotschy and Sunde (2018) and Kotschy et al. (2020) demonstrate that aging can cause some pressures on the macroeconomic performance and that the age structure of the labor force is decisive for development. According to the authors, education eases, but does not completely compensate for the impacts of demographic changes, unless it is marked by a continuous process of expansion, particularly in developing countries.

Future tendencies of increasing levels and quality of schooling can potentially mitigate the consequences of diminishing growth rates of the working‐age population. It is observed a positive correlation between increased levels of schooling and participation in the labor force, particularly of women and older adults (55+) (Miller, Saad, and Martinez, 2016; Loichinger and Prskawetz, 2017; Matsukura et al. 2018; Scott, 2023). Investments in human capital are associated with higher productivity and increased wages (Becker, 2007; Weiss, 2015). More educated individuals have access to better job opportunities and tend to be more productive. Given the strong link between health and education, expanding educational attainment within a population can also enhance the duration of working life.

Older workers tend to stay in the labor force when they are more qualified and have high status occupations. The potential increase in the number of the elderly in the labor force reduces dependency levels, constituting a silver dividend (Fernandes and Queiroz, 2024; Matsukura et al. 2018). This phenomenon is better suited when followed by other socioeconomic improvements such as increasing education, reducing inequalities, discrimination and informality, and better health (Scott, 2023). However, it is essential to adopt a life‐cycle perspective, that is, educational investments should be made early in life to yield optimal returns. Investing in education at older ages may yield lower‐than‐expected benefits due to the shorter period for returns (Maestas and Zissimopoulos, 2010; Scott, 2021).

While demographic changes take place, other social and institutional aspects also arise and could balance the potential problems that the aging process causes on economy. While changes in population age structure undeniably influence economic outcomes, the literature suggests that education has an even greater impact. This is evident in a series of developing countries, where improvements in educational attainment can significantly offset the economic pressures of rapid aging. These insights call for a shift in focus from merely managing demographic transitions to actively enhancing human capital.

Beyond its economic impacts, education profoundly influences individual and societal well‐being (Fernandes and Queiroz, 2024; Lutz et al. 2019). Higher education levels are associated with better health outcomes, increased civic engagement, and greater social cohesion. As pointed out by Timmis and Hallsworth (2024), educational expansion is pivotal to promote balanced interactions between humanity and other beings that live on the planet. It must be considered of interest of a broad spectrum of agents—academia, governments, public and private institutions, and other individuals—once education is capable of amalgamate a set of factors that lead to sustained development and contribute to the overall stability and prosperity of societies, particularly those undergoing demographic transitions. Recent research shows that education leads to higher labor force participation at all ages, although it depends on how young or old a population is. If all individuals had the returns of the more educated, the levels of support would be higher, and societies would have more time to capitalise the demographic dividends, particularly developing countries (Fernandes and Queiroz, 2024).

As global demographic transitions continue to unfold, the importance of education will only grow. It is central that policymakers recognise its transformative potential and act decisively to secure a future of prosperity and well‐being. However, educational dividends are not automatic and depend on institutions and policies to transform changes in education and population age structure into economic development and growth. For instance, it is fundamental that the labor market creates enough opportunities for the growing working age population and to absorb older adults that could remain in the labor force for longer periods of time. Improvements in education must reach the whole population, not only in education attainment, but also in the quality of education.

## Author Contributions

**Anderson Rocha de Jesus Fernandes:** conceptualization, writing – original draft, writing – review and editing. **Bernardo Lanza Queiroz:** validation, writing – original draft, writing – review and editing.

## Conflicts of Interest

The authors declare no conflicts of interest.

## Data Availability

Data sharing not applicable to this article as no datasets were generated or analysed during the current study.
